# Insensible Is a Novel Nuclear Inhibitor of Notch Activity in *Drosophila*

**DOI:** 10.1371/journal.pone.0098213

**Published:** 2014-06-05

**Authors:** Franck Coumailleau, François Schweisguth

**Affiliations:** 1 Institut Pasteur, Developmental & Stem Cell Biology Dept, Paris, France; 2 CNRS, URA2578, Paris, France; National Institutes of Health (NIH), United States of America

## Abstract

Notch signalling regulates a wide range of developmental processes. In the *Drosophila* peripheral nervous system, Notch regulates a series of binary fate decisions that lead to the formation of regularly spaced sensory organs. Each sensory organ is generated by single sensory organ precursor cell (SOP) via a series of asymmetric cell divisions. Starting from a SOP-specific Cis-Regulatory Module (CRM), we identified *insensible (insb)*, a.k.a CG6520, as a SOP/neuron-specific gene encoding a nuclear factor that inhibits Notch signalling activity. First, over-expression of Insb led to the transcriptional repression of a Notch reporter and to phenotypes associated with the inhibition of Notch. Second, while the complete loss of *insb* activity had no significant phenotype, it enhanced the bristle phenotype associated with reduced levels of Hairless, a nuclear protein acting as a co-repressor for Suppressor of Hairless. In conclusion, our work identified Insb as a novel SOP/neuron-specific nuclear inhibitor of Notch activity in *Drosophila*.

## Introduction

Cell fate decisions and patterning events during development are regulated by cell-cell interactions that are in part mediated by Notch receptors [1]. Trans-membrane receptors of the Notch family can be described as membrane-tethered transcriptional regulators [2]. Indeed, these receptors consist in a ligand-binding ectodomain linked via a trans-membrane domain to an intracellular domain that acts as a transcriptional regulator upon its ligand-dependent release from the membrane. A ligand-dependent conformational change in the ectodomain of Notch is thought to result in ectodomain shedding and intra-membrane processing of Notch. Following the release of the Notch Intra-Cellular Domain (NICD), the activated nuclear form of Notch [2], NICD forms a ternary complex with CSL (CBF1, Suppressor of Hairless, Lag-1), a sequence-specific DNA-binding protein known as Suppressor of Hairless [Su(H)] in flies, and a co-activator, known as Mastermind (Mam) in flies, to regulate the expression of Notch target genes. In the absence of NICD, CSL factors can bind the *cis* regulatory region and repress the expression of a subset of Notch target genes in both flies [3], [4], [5], [6] and mammals [7], [8], [9], [10]. Indeed, the human CSL factor CBF1 was initially identified as a transcriptional repressor [11] and several different CSL co-repressors have been identified in mammalian cells [7], [8], [12], [13]. NICD increases the occupancy of CSL binding sites, relieves the transcriptional repression mediated by CSL factors and promotes transcriptional activation [2], [3], [9], [14].

In *Drosophila*, repression by Su(H) is critical to prevent Notch target genes from being inappropriately activated in some developmental contexts [3], [4], [5]. Su(H) acts in part by recruiting the adaptor protein Hairless (H) and its co-repressors CtBP and Groucho [15]–[20]. While the activity of H appeared to be dispensable in most developmental contexts [17], including embryogenesis [21], repression by Su(H)-H complexes is required for cell fate decisions during adult peripheral neurogenesis [15], [17]–[20], [22]. During pupal development, the activity of *H* is first required in imaginal tissues for the stable determination of Sensory Organ Precursor cells (SOPs). SOP specification relies on Notch-mediated lateral inhibition such that Notch target genes are repressed in SOPs (Notch OFF) and activated in surrounding cells (Notch ON). The de-repression of Notch target genes in *H* mutant SOPs was shown to prevent their stable determination [5], [22]. Following their specification, each SOP undergoes a stereotyped series of asymmetric cell divisions to generate the four different cells forming a sensory bristle. The activity of *H* is also required for proper cell fate determination in the bristle lineage. A reduced level of *H* in heterozygous or hypomorphic mutant flies led to the transformation of shaft into a second socket, hence resulting into double-socket bristles [17].

Repression by Su(H)-H complexes may act in parallel to other regulatory mechanisms to inhibit the expression of Notch target genes in SOPs. For instance, the transcriptional repressor Longitudinal lacking (Lola) was shown to repress the expression of Notch target genes [23], and to genetically interact with *H* during adult peripheral neurogenesis [24]. Additionally, the nuclear BEN-solo family protein Insensitive (Insv) was recently shown to directly interact with Su(H) and to inhibit in a *H*-independent manner the expression of Notch target genes, both in embryos and in a cell-based assay [25]. This CSL co-repressor activity appears to be conserved in mammals since BEND6, a mouse homolog of Insv, binds CSL and antagonizes Notch-dependent gene expression in neural cells [26]. Of note, both Lola and Insv appear to be expressed at higher levels in SOPs, indicating that repression of Notch target genes is achieved by several mechanisms in this developmental context.

In this study, we characterized the genetic function of the *CG6520/insb* gene that encodes a novel SOP/neuron-specific nuclear protein involved in the repression of Notch target genes.

## Results

### Insensible is a SOP-specific Nuclear Protein

**Figure 1:**
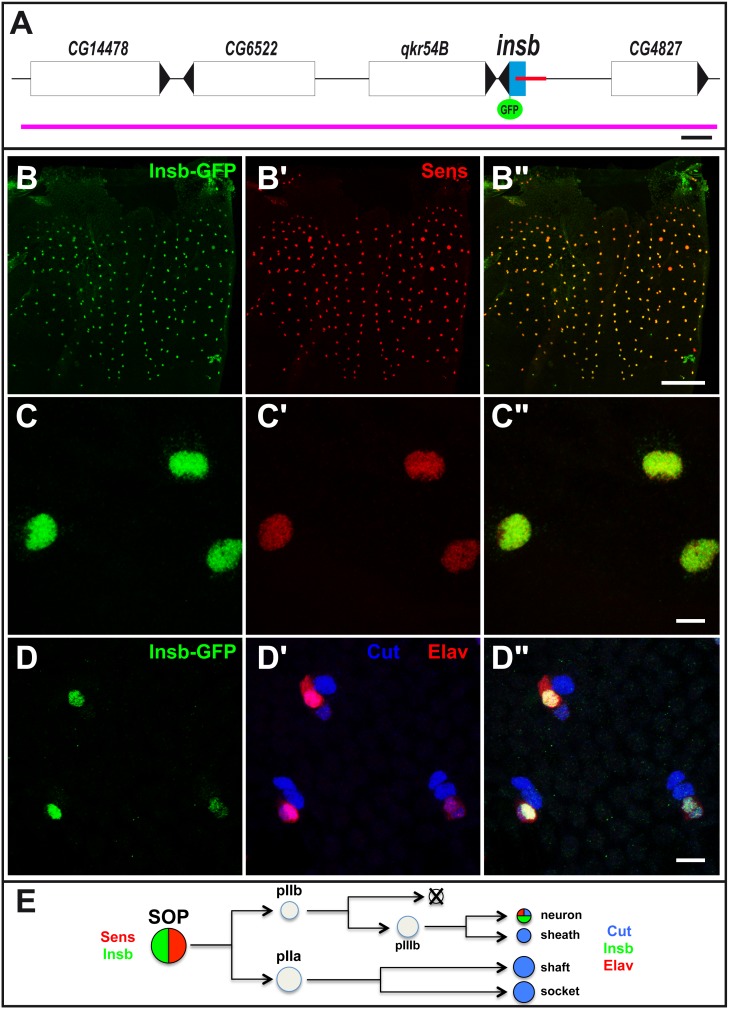
*insb* encodes a nuclear SOP-specific protein. (A) Schematic representation of the *insb* genomic region: genes positions and orientations are shown in white with the exception of *insb* (blue). The SOP-specific CRM [24] is shown in red. The BAC used in this study is indicated in purple. GFP (green) was inserted 3′ to the *insb* ORF. Scale bar is 1 kb. (B–C”) Insb-GFP (GFP, green) was specifically detected in the nucleus of SOPs, marked by Senseless (Sens, red), in the notum of 16 hrs after puparium formation (APF) pupae. (D–D”) Insb-GFP (GFP, green) was detected in neurons (Elav, red) but not in other sensory organ cells (Cut, blue) at 24 hr APF. (E) Diagram of the bristle lineage with the markers used in this study. Scale bars are 100 µm. (B–B”) and 5 µm. (C–D”).

In a previous study, we used an *in silico* approach to identify *cis*-regulatory modules (CRM) regulating gene expression in sensory organ precursor cells (SOPs) in *Drosophila* [24]. This work led to the identification of a CRM just 5′ to the gene *CG6520/insensible* (*insb*) (Figure 1A) which was active in SOPs and other neural progenitor cells. The function of the gene *insb* is not known in *D. melanogaster* and orthologs of *insb* could only be identified in invertebrates. This gene encodes a novel small protein of 176 amino acids with no clear sequence similarities with previously described proteins and/or domains. Sequence analysis suggested the existence of a conserved bipartite nuclear localization signal and of two short motifs that are conserved amongst *Drosophilidae* orthologs (Figure S1).

To test whether *insb* is expressed in SOP cells, we generated a GFP tagged-version of Insb expressed under its own regulatory sequences. Starting from a 22 kb genomic BAC covering the *insb* locus, we used recombineering in *E. coli* to generate an Insb-GFP BAC transgene (Figure 1A) [27], [28]. Analysis of endogenous Insb-GFP BAC expression in transgenic flies indicated that the *insb* gene was expressed in SOPs of the pupal notum (Figure 1B–B”). Additionally, we observed that the Insb-GFP protein was nuclear (Figure 1C–C”). Following the division of SOPs, Insb-GFP was detected in pIIa and pIIb cells and is later restricted to neurons (Figure 1D–E) (see [29] for a description of the bristle lineage). Consistent with these observations, RNA-seq data indicated that *insb* transcripts were specifically detected in the nervous system both during development and in the adult [30]. We conclude that *insb* is a SOP/neuron-specific gene that encodes a novel nuclear protein.

### *Insensible* is not an Essential Gene

**Figure 2:**
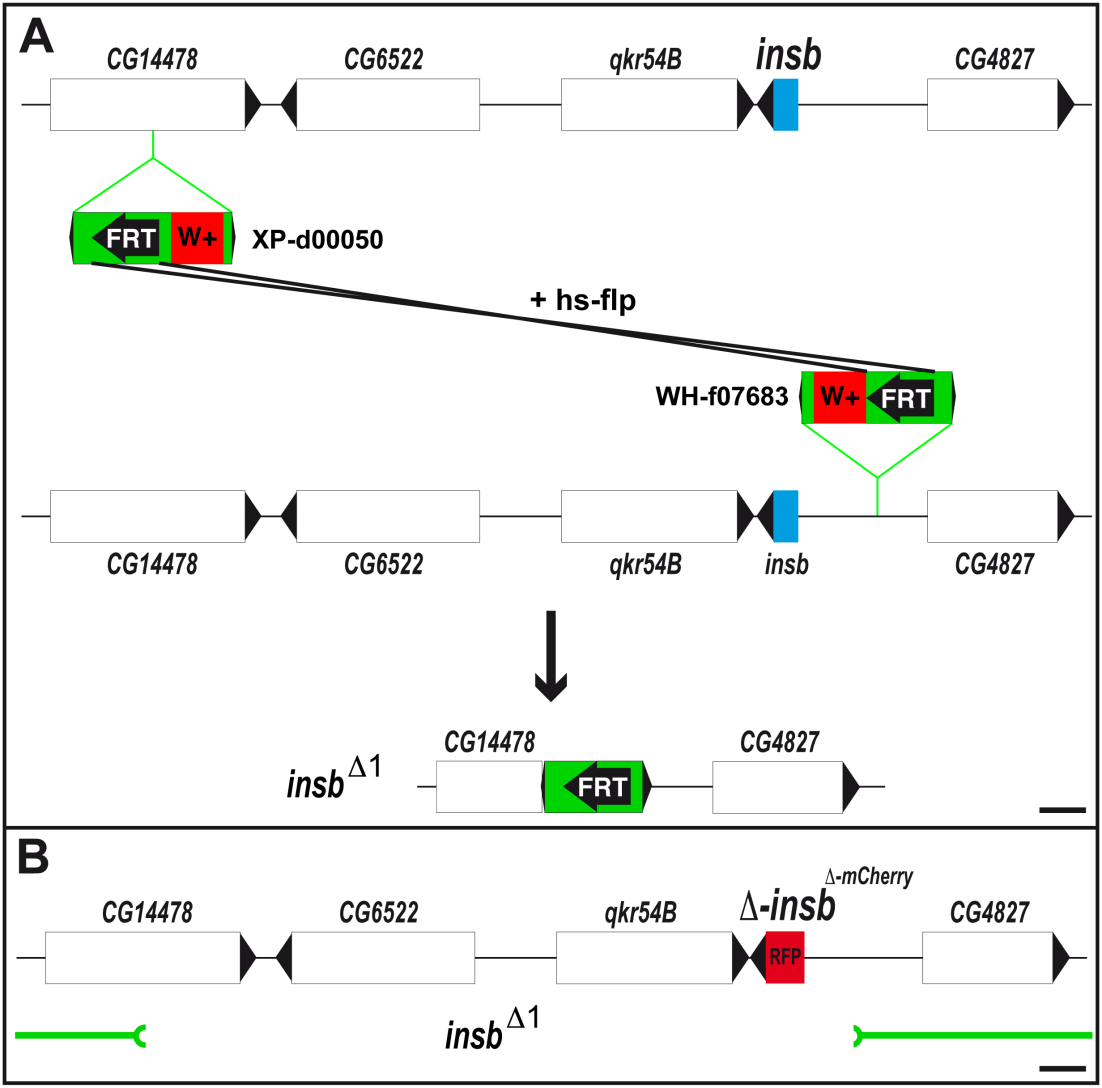
Generation of a synthetic *insb* null allele. (A) Generation of the *insb* ^Δ1^ deficiency using Flp-FRT recombination between the two chromosomes carrying the P{XP}d00050 and PBac{WH}f07683 insertions. (B) Schematic representation the *insb^mCherry^* BAC used for the genomic rescue of the *CG14478*, *CG6522* and *qkr54B* genes deleted by *insb* ^Δ1^ deficiency (shown in green below). The ORF of *insb* was replaced by the *mCherry* encoding sequence (RFP, red). Scale bars are 1 kb.

To study the function of the *insb* gene, we first used a loss of function approach. Since no mutation was available for this gene, we generated a deletion covering the *insb* gene. To generate a small deletion with precisely defined breakpoints, we took advantage of transposon insertion lines containing FRT sites [31]. Briefly, we used two FRT-containing transposons located 5′ (XP-d05000) and 3′ (WH-f07683) of the *insb* gene to select a *trans*-recombination event resulting in a 15 kb genomic deletion (*insb* ^Δ*1*^; Figure 2A). This deletion removed the *CG14478*, *CG6522*, *qkr54B* and *insb* genes (Figure 2B). Homozygous *insb* ^Δ*1*^ flies were viable and fertile. We therefore conclude that the activity of the *insb* gene is largely dispensable for fly development. Of note, the *CG14478*, *CG6522*, *qkr54B* genes similarly appeared to be dispensable for viability in the context of the laboratory.

To generate a single mutant background, i.e. mutant only for the *insb* gene, we rescued the *insb* ^Δ*1*^ deletion allele by a BAC transgene encoding the *CG14478*, *CG6522*, *qkr54B* genes and in which the open reading frame (ORF) of *insb* was replaced by those of *mCherry* (Figure 2B). This mutant BAC is referred to here as *insb^mCherry^* and the synthetic combination of the *insb* ^Δ*1*^ deletion with the *insb^mCherry^* BAC transgene as *insb^m^*.

**Figure 3:**
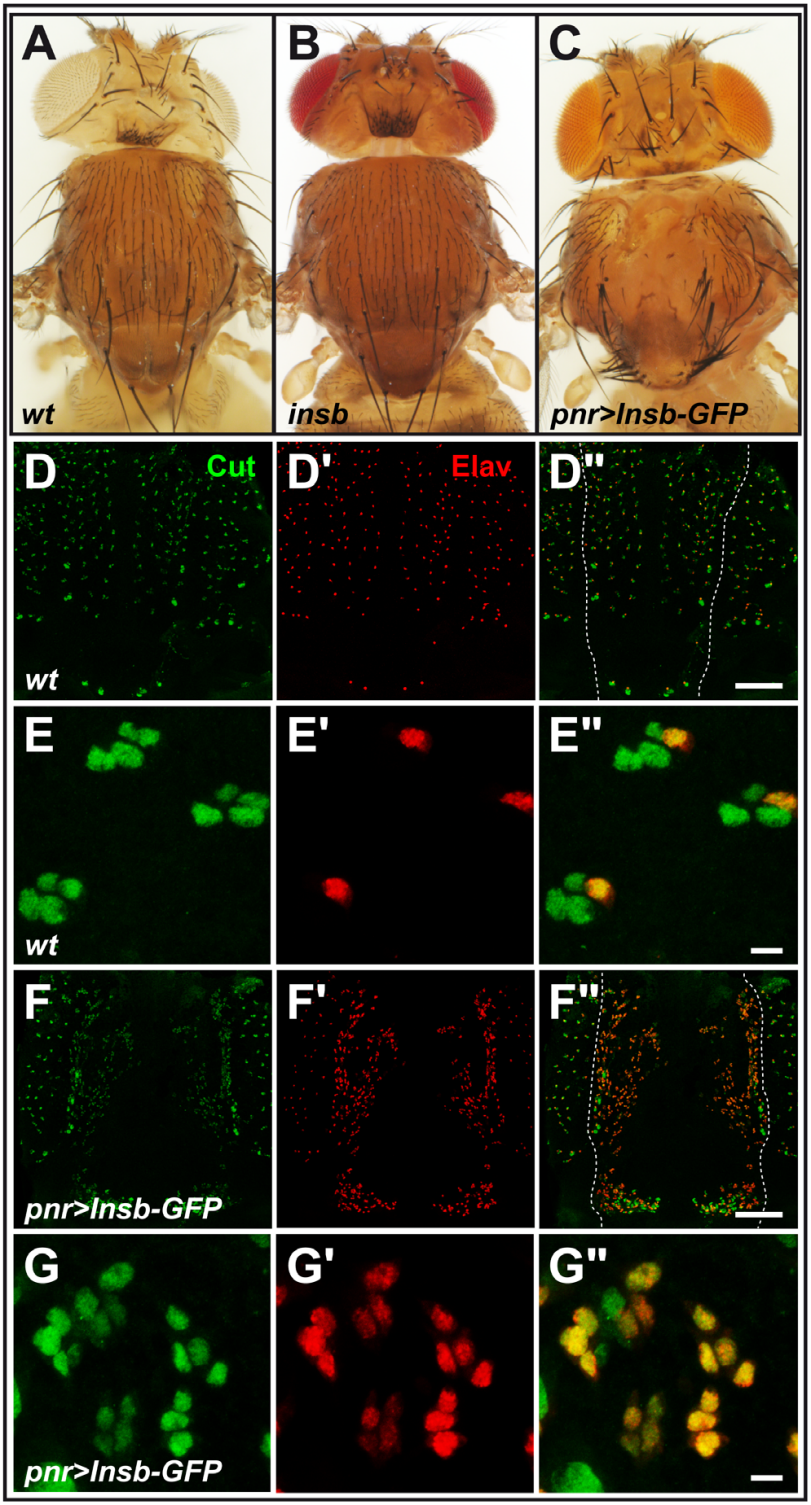
Overexpression of Insb led to Notch inhibition. (A–C) micrographs showing the bristle pattern on the dorsal thorax of control (A), *insb^m^* (synthetic mutation resulting from combining the *insb* ^Δ1^ deficiency with the *insb^mCherry^* BAC; B) and *pnr>Insb-GFP* (C) flies. Loss of *insb* had no significant effect on the bristle pattern whereas ectopic expression led to bristle loss (associated with a transformation of external cells into internal cells) and bristle tufts (due to an excess of SOPs). (D–G”) The bristle loss phenotype of *pnr>Insb-GFP* flies was associated with i) an increased density of sensory organs (Cut, green; Elav, red) in the dorso-central region (bracket) of the notum in 22 hrs APF pupae (compare *pnr>Insb-GFP* pupae in F–F” with wild-type (wt) pupae in D–D”) and ii) a transformation of sense organ cells into neurons (Elav, red). Both increased density of sensory organs and transformation of sensory cells into neurons are indicative of a strong loss of Notch signalling (see magnifications in E–E” and G–G”). The expression pattern of *pnr-Gal4* is indicated with dashed lines in D” and F”. Scale bars are 100 µm. (D–D” and F–F”) and 5 µm. (E–E” and G–G”).

Adult *insb^m^* flies showed no clear developmental defects with sensory bristles showing a normal pattern on the body surface (Figure 3A, B). This indicated that the specification of SOPs was largely unaffected by the complete loss of *insb* activity. Additionally, no significant bristle phenotype was observed at macrochaete positions in *insb^m^* mutant flies (Figure 3B; see also below the phenotype of flies heterozygous for *insb^m^* over a deficiency). Thus, this loss-of-function analysis showed that *insb* is not an essential gene.

**Table 1:** Genotypes.

Figures	Genotypes
Fig. 1B–D”	*w/w;; PBac{y[+]-attP-9A. insb^GFP^}VK00019*
Fig. 3A, 3D–E”	*w/w*
Fig. 3B	*w/w; FRT(w[hs])}G13, insb* ^Δ*1*^ */FRT(w[hs])}G13, insb* ^Δ*1*^ *; PBac{y[+]-attP-9A. insb* ^Δ*mCherry*^ *}VK00019/PBac{y[+]-attP-9A. insb* ^Δ*mCherry*^ *}VK00019*
Fig3C, 3F–3G”	*w/w;; pnr-Gal4/UAS-Insb-GFP*
Fig. 4A–A”	*w/w; en-Gal4, Gal80^ts^/+*
Fig. 4B–B”	*w/w; en-Gal4, Gal80^ts^/UAS-Insb-GFP*
Fig. 5A, I	*w/w*
**Fig. 5B, I**	*w/w; FRT(w[hs])}G13, insb* ^Δ*1*^ */Df(2R)BSC406; PBac{y[+]-attP-9A. insb* ^Δ*mCherry*^ *}VK00019/+*
Fig. 5C, I	*w/w; insv^23B^/insv^23B^*
Fig. 5D, I	*w/w; FRT(w[hs])}G13, insb* ^Δ*1*^ *, insv^23B^/FRT(w[hs])}G13, insb* ^Δ*1,*^ * insv^23B^; PBac{y[+]-attP-9A. insb* ^Δ*mCherry*^ *}VK00019/TM6B, Tb* ^[*1*]^
Fig. 5E, I	*w/w;; H^[E31]^, P[mw+], P[neo, FRT]82B/TM6B, Tb* ^[*1*]^
Fig. 5F	*w/w; FRT(w[hs])}G13, insb* ^Δ*1*^ */FRT(w[hs])}G13, insb* ^Δ*1*^ *; PBac{y[+]-attP-9A. insb* ^Δ*mCherry*^ *}VK00019, H^[E31]^/TM6B, Tb* ^[*1*]^
Fig. 5G, I	*w/w; insv^23B^/insv^23B^; H^[E31]^, P[mw+], P[neo, FRT]82B/TM6B, Tb* ^[*1*]^
Fig. 5H, I	*w/w; FRT(w[hs])}G13, insb* ^Δ*1*^ *, insv^23B^/FRT(w[hs])}G13, insb* ^Δ*1,*^ * insv^23B^; PBac{y[+]-attP-9A. insb* ^Δ*mCherry*^ *}VK00019, H^[E31]^/TM6B, Tb* ^[*1*]^
**Fig. 5I**	*w/w; FRT(w[hs])}G13, insb* ^Δ*1*^ */Df(2R)BSC406; PBac{y[+]-attP-9A. insb* ^Δ*mCherry*^ *}VK00019, H^[E31]^/+*
Fig. 5**I**	*w/w; FRT(w[hs])}G13, insb* ^Δ*1*^ */Df(2R)BSC406; PBac{y[+]-attP-9A. insb* ^Δ*mCherry*^ *}VK00019, H^[E31]^/PBac{y[+]-attP-9A. insb^GFP^}VK00019*
Fig. 6A–B”	*w/w; insv^23B^/insv^23B^; PBac{y[+]-attP-9A. insb^GFP^}VK00019/PBac{y[+]-attP-9A. insb^GFP^}VK00019*
Fig. 6C–C”	*w/w; en-Gal4, Gal80^ts^/+; PBac{y[+]-attP-9A. insb^GFP^}VK00019/UAS-insv*
Fig. 6D	*w/w, sd-Gal4, Gal80^ts^*
Fig. 6E	*w/w, sd-Gal4, Gal80^ts^;; UAS-Insb-GFP/+*
Fig. 6F	*w/w, sd-Gal4, Gal80^ts^; insv^23B^/insv^23B^; UAS-Insb-GFP/+*

### *Insensible* can Inhibit the Expression of Notch Target Genes

We next used a gain-of-function approach to further examine the function of the *insb* gene. To do so, we generated a UAS-Insb-GFP transgene. The ectopic expression of the Insb-GFP protein was then achieved using various Gal4 drivers. Using *pannier-Gal4* (*pnr-Gal4*), we found that over-expression of Insb-GFP resulted in a bristle loss phenotype (Figure 3C). This balding phenotype was associated with both an increased density of sensory organs in the dorso-central region of the notum that expressed *pnr-Gal4* (Figure 3D–D” and F–F”) and an increased number of Elav-positive neurons (Figure 3E–E” and G–G”). Hence, we propose that overexpression of Insb-GFP led first to the specification of too many SOPs, hence the increased number of external sensory organs, and second to the transformation of external cells into internal cells, notably neurons, leading to the balding phenotype seen in adult flies. Thus, this *insb* gain-of-function generated a lateral inhibition and cell fate transformation phenotypes similar to the ones observed upon loss of Notch activity [32]. We therefore propose that the *insb* gene encodes a nuclear antagonist of Notch.

**Figure 4:**
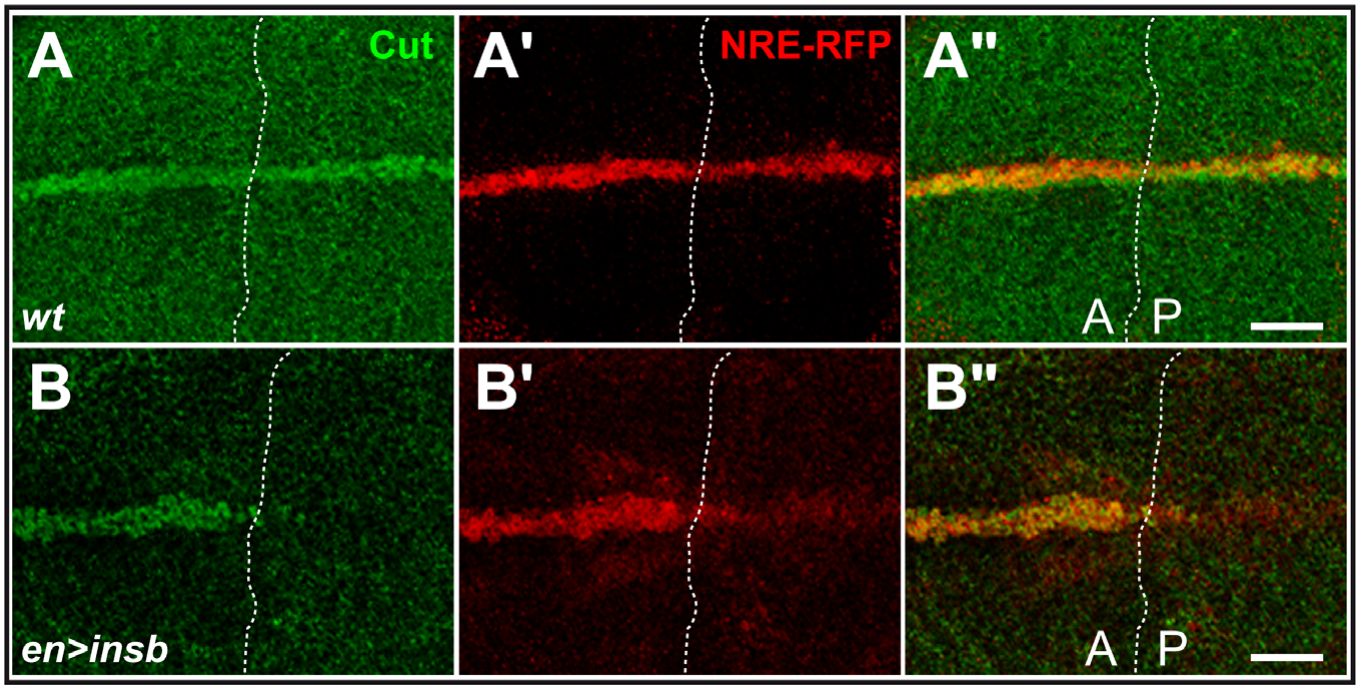
Ectopic Insb inhibits the expression of Notch targets. (A) Expression of Insb in posterior (P) cells led to the reduced expression of the Notch target gene *cut* (Cut, green) and of the NRE-RFP reporter (RFP, red) in third instar wing imaginal discs (B–B”; compare with a wt control disc showing the expression of Cut and NRE-RFP in both anterior (A) and P cells along the dorsal-ventral boundary in A–A”). Scale bars are 20 µm. (A–B”).

To further test this proposal, we analysed the effect of *insb* over-expression on another Notch-dependent process. The development of the wing involves the activation of Notch at the wing margin. We found that ectopic expression of Insv-GFP in posterior cells using the *en-Gal4* driver inhibited the expression of the Notch target gene *cut* (Figure 4) [33] and resulted in posterior notches in adult wings (not shown). Moreover, the expression of an artificial Notch reporter construct, NRE-RFP [34], was also significantly reduced in posterior cells (Figure 4). We therefore conclude from these gain-of-function experiments that *insb* can inhibit the activity of Notch. We therefore propose that Insb is a nuclear antagonist of Notch that contributes to inhibit the expression of Notch target genes in SOPs (and more generally in neural cells).

### *Insensible* Genetically Interacts with *Hairless*

**Figure 5:**
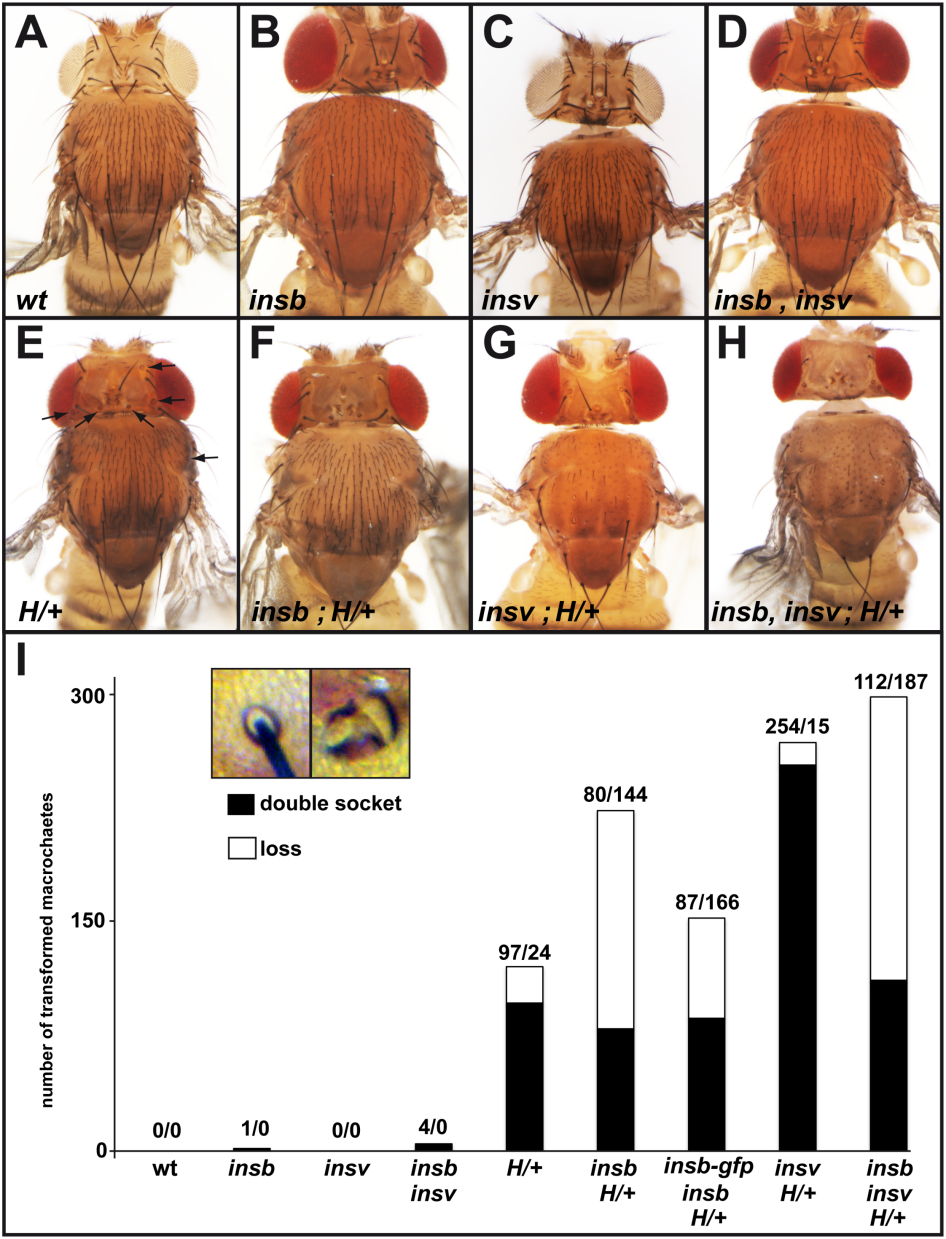
*insb* genetically interacts with *H.* (A–H) Micrographs showing the bristle pattern on the dorsal thorax of control (A), *insb^m^*/ ,*Df(2R)BSC406* (B), *insv^23B^* (C), *insb^m^ insv^23B^* (D), *H^E31^/^+^* (E) (affected macrochaetes are indicated with an arrow), and *insb^m^*, *H^E31^/H^+^* (F), *insv^23B^*, *H^E31^/^+^* (G), *insb^m^ insv^23B^ H^E31^/^+^* (H) flies (see Table 1 for detailed genotypes). (I) Histogram showing the number of lost (no shaft, no socket) and double-socket (no shaft) macrochaetes in the genotypes shown in A–H (for each genotype, 10 animals were scored for a total of 400 macrochaetes). A strong genetic interaction was observed between *insb* and *H*. The inset shows a control and a double-socket bristle from an *H^E31^/^+^* fly.

Several nuclear factors are known to contribute to the repression of Notch target genes in SOPs, including H [19], [35] [17] and Insv [25]. To further test the role of *insb* in the regulation of Notch target genes, we first studied genetic interaction between *insb* and *H*. To do so, we counted the number of missing and double-socket macrochaetes in flies mutant for *insb^m^* over a 55 kb deficiency deleting the *insb* locus, *Df(2R)BSC406,* in flies heterozygous for *H^E31^*, a null allele of *H*. As reported earlier, we found that heterozygous *H^E31^* mutant flies exhibited a mild double-socket phenotype and a weak bristle loss phenotype (Figure 5E, I). Loss of *insb* in this context enhanced the bristle loss phenotype (Figure 5F, I). Moreover, the Insb-GFP BAC suppressed this genetic interaction. We conclude that *insb* genetically interacts with and that the BAC-encoded Insb-GFP is functional. Earlier studies had shown that the *H* bristle loss phenotype resulted from a failure of SOP determination due to a defect in the repression of Notch target genes in SOPs [35]. Thus, this genetic interaction was consistent with *insb* contributing to the repression of Notch target genes in SOPs. It further indicated that this proposed function of *insb* becomes essential when the activity of *H* is limiting. Consistent with a role of *insb* in antagonizing Notch signaling, we also found that the loss of *insb* activity suppressed the bristle density phenotype of *Notch* heterozygous flies (*N^55e11^*/+: 153+/−9 bristle in the dorso-central region; *N^55e11^*/+; *insb* ^Δ*1*^ */Df(2R)BSC406*: 133+/−7; n = 10 flies; wild-type and *insb* mutant flies had 120+/−4 and 128+/−6, respectively).

We next tested interaction between *insb^m^* and *insv^23B^*, a null allele of *insv* [25]. As reported earlier, *insv* mutant flies had no detectable macrochaete bristle phenotype (Figure 5C, I) [25]. We also confirmed that the loss of *insv* activity strongly enhanced the *H* double socket phenotype (Figure 5G, I) [25]. While no interaction was observed in *insb insv* double mutant flies (Figure 5D, I), the loss of *insb* enhanced the bristle loss phenotype of *insv H^+/−^* flies (Figure 5H, I). One possible interpretation for these genetic interaction data is that the nuclear factors Insb and Insv act together, in parallel with H, to inhibit Notch target gene expression in SOPs and its progeny cells.

**Figure 6:**
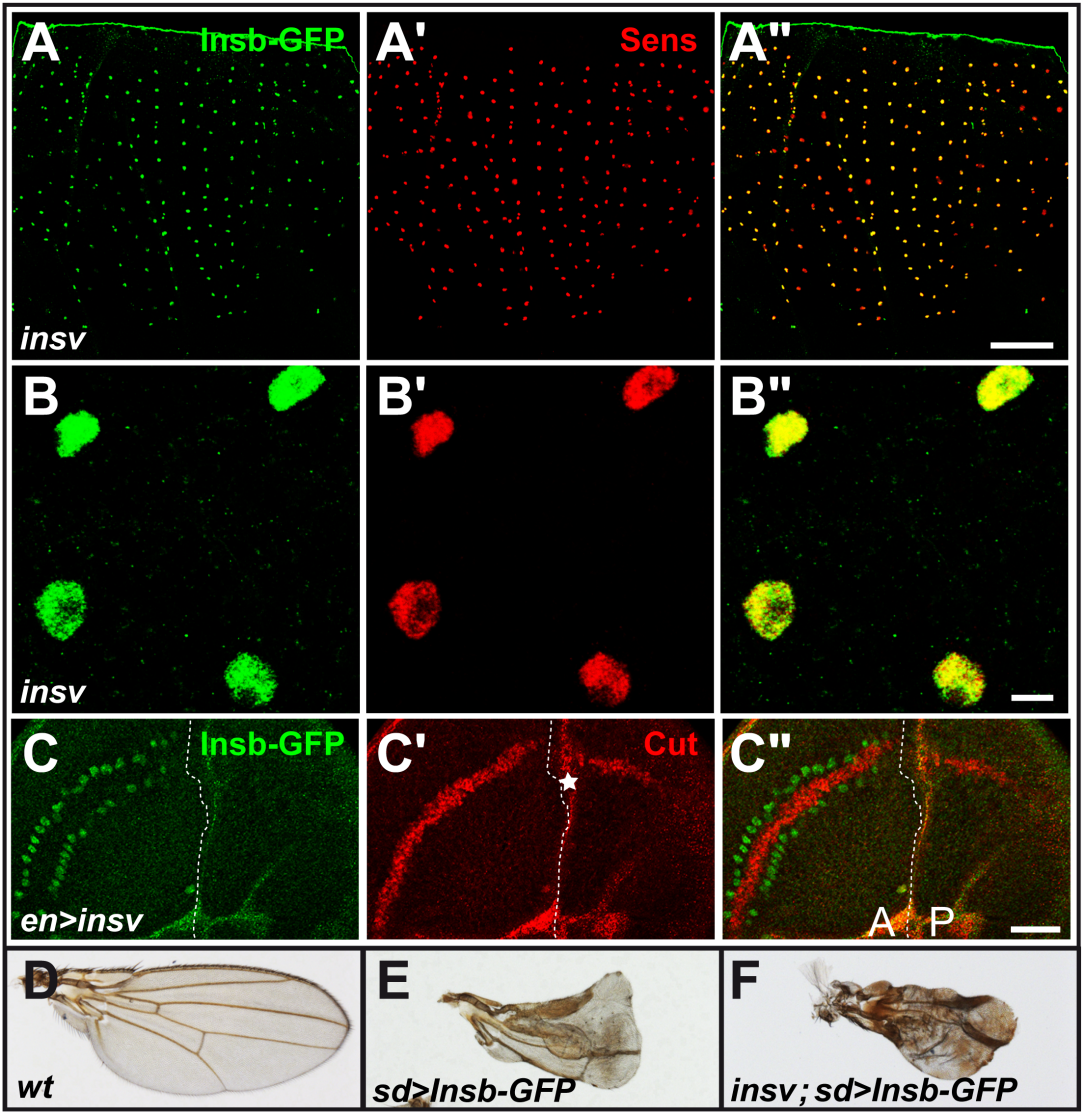
Insv and Insb may act independently of one another. (A–B”) Insb-GFP (GFP, green) was specifically detected in the nucleus of SOPs (Sens, red), in 16 hrs APF *insv* mutant pupae. (C–C”) Ectopic expression of Insv in posterior (P) cells inhibited the expression of the Notch target gene *cut* (Cut, red) in wing discs without inducing the expression of Insb-GFP (GFP, green). The background red signal (star in C’) was generated by image projections due to a fold along A/P boundary in wing discs over-expressing Insv. (D–F) adult wings: wild-type (D), overexpression of Insb-GFP in wild-type (E) and *insv* mutant background (F) using *sd-Gal4* driver. Loss of *insv* function had no effect on the Insb-GFP induced phenotype. Scale bars are 100 µm (A–A”), 5 µm (B–B”) and 20 µm (C–C”).

To begin testing whether *insb* and *insv* act within in a linear regulatory pathway, we examined the expression of the *insb-GFP* BAC transgene in *insv* mutant flies. We found that that the *insb-GFP* gene was normally expressed in SOPs (Figure 6A–A”) and that the Insb-GFP protein was still nuclear (Figure 6B–B”), We conclude that the SOP-specific expression of *insb* and the nuclear localization of Insb did not depend on *insv* activity. Additionally, ectopic expression of *insv* in wing imaginal discs using *engrailed-Gal4* (*en-Gal4*) led to a loss of *cut* expression in posterior cells (Figure 6C–C”) and to a wing notching phenotype in adult flies (data not shown) but did not result in ectopic *insv-GFP* expression (Figure 6C). These data did not support a model whereby Insv regulates the expression of *insb*. It also suggested that repression of Notch target genes by Insv can take place without up-regulating the expression of *insb*.

We next tested whether Insb can inhibit the activity of Notch in the absence of *insv*. To do so, Insb-GFP was overexpressed using *sd-Gal4* in wing imaginal discs of wild-type and *insv* mutant flies. We found that the Notch-like phenotypes induced by overexpressed Insb-GFP, i.e. loss of wing margin, was not suppressed by the loss of *insv* activity (Figure 6D–F). This result suggested that Insb can inhibit Notch independently of Insv. Together, our data suggest that repression of Notch targets by Insb is important when the level of H is limiting and that Insv may function independently of Insb to inhibit the expression of Notch target genes.

## Discussion

Our study identified Insb as a novel SOP/neuron-specific nuclear factor that antagonizes Notch to regulate cell fate. First, we have shown that over-expression of Insb inhibited the activity of Notch during sensory organ formation and blocked the expression of a Notch reporter construct in wing discs. This indicated that Insb has the ability to inhibit the expression of Notch target genes. Since the Notch reporter construct used here responded directly to Notch via paired Su(H) binding sites [34], [36], [37], Insb likely acts via these binding sites, i.e. by modulating the activity of Su(H)-bound complexes. Second, while the activity of *insb* appeared to be largely dispensable during development, its activity became essential for the proper determination of sensory bristle cells when the activity of *H* becomes limiting, i.e. when Notch target genes are derepressed upon reduced H levels [22]. Thus, like Insv, Insb appears to function in a partly redundant manner with H. Additionally, while loss of *insb* and *insv* activities similarly enhanced the *H* haplo-insufficient phenotype, no genetic interaction was observed in double mutant flies. One possible interpretation for this lack of genetic interaction is that Insv and Insb act together to regulate the same process, so that the complete loss of one or both genes have similar phenotypic consequences. Since Insv did not regulate the expression of *insb*, one possibility is that Insb positively regulates the expression of the *insv* gene and that Insv antagonizes Notch. Alternatively, the two proteins may act together to repress the expression of Notch target genes via the Su(H) binding sites. Consistent with this, Insv was proposed to repress the expression of Notch target genes by two mechanisms: first in a Su(H)-dependent mechanims, Insv would act as a CSL co-repressor to promote repression through Su(H) binding sites; second, Insv may directly bind DNA via its BEN domain and regulate gene expression in a Su(H)-independent manner. Whether Insb physically interacts with Insv and regulates its transcriptional activities await biochemical studies. While a functional homolog of Insv has recently been characterized in the mouse, no clear homolog of Insb could be easily identified in vertebrates. Thus, deciphering how Insb regulates in flies the activities of Insv and other CSL associated co-repressors, such as H, may provide new insights into molecular mechanisms of co-repression by CSL-associated factors. Finally, while the expression and function of Insb was primarily studied here in the context of sensory organ development, this gene was also expressed at high levels in neuroblasts of the developing larval brain, suggesting that Insb may have a broader role as a Notch antagonist.

In conclusion, our study identified Insb as a nuclear SOP/neuron-specific antagonist of Notch signaling that may act together with Insv to repress the expression of Notch target genes.

## Materials and Methods

### Flies and Transgenes

The *insb* ^Δ1^ deficiency was generated by Flp-FRT recombination as described in Parks *et al,* [31] using P{XP}d00050 and PBac{WH}f07683. The resulting 15,396 nt deletion (corresponding to deficiency *FDD-0000787* in http://www.drosdel.org.uk/fdd/fdd_info.php) was selected based on eye color (loss of *w+*) and confirmed by PCR.

The CH322-168B11 BAC covering the *insb* locus (from 6,148 nt 5′ to the transcription start site to 15,129 nt downstream of the 3′UTR) was obtained from BACPAC (http://bacpac.chori.org) [28] and used to generate the *insb-GFP*, and *insb* ^Δ*mCherry*^ transgenes by BAC recombineering in *E. coli* as described in [27]. The 5′ and 3′ homology arms were produced using the following primers:

For the *insb-GFP* allele;

CG6520_5F: GCAACCGACTGAAGCGGTTCCGGA

CG6520_Rgfp: CAAAAACACCCCGCCCTAACAACA

CG6520_Fgfp: CTGTACAAGTAAGAGCAAACCGGAGGGCAG

CG6520_3R: CTTGCTCACCATGGCGTGCAGAAGTCCATC

The GFP cassette was amplified using the following primers:

CG6520_GfpF: CTTCTGCACGCCATGGTGAGCAAGGGCGAG

CG6520_GfpR: TCCGGTTTGCTCTTACTTGTACAGCTCGTC

For the *insb* ^Δ*mCherry*^ allele;

CG6520_5Fch: GACGGATGCACGGAGGAAGGGA

CG6520_Rch: CTCCTCGCCCTTGCTCACCATGCTTAGAGGATGAT

CG6520_Fch: CTGTACAAGTAAGAGCAAACCGGAGGGCAGGA

CG6520_3Rch: CTTGCTCACCATGGCGTGCAGAAGTCCATC

The mCherry cassette was amplified using the following primers:

CG6520_chF: CCTCTAAGCATGGTGAGCAAGGGCGAGGA

CG6520_chR: TCCGGTTTGCTCTTACTTGTACAGCTCGTC

Constructs were verified by sequencing of the recombined regions prior to phiC31-mediated integration at the PBac{y[+]-attP-9A}VK00019 site [27].

The UAS-Insb-GFP transgene was generated by PCR amplifying the ORF of CG6520 from wild-type genomic DNA using the primers;

5′-CG6520: CGGGATCCATGTCGGGCAAACTGATCATGA

3′-CG6520: GGGGTACCGGGGCGTGCAGAAGTCCATCGCT

The PCR product was cloned as a BglII/KpnI fragment into pUAST-EGFP. After verification by sequencing, the transgene was inserted into the fly genome by P-element transformation.

All injections were performed by BestGene Inc. (Chino Hills, USA).

The *Df(2R)BSC406*, *H^E31^* [21], *insv^23B^* and UAS-*insv* flies [25] have been described previously. Conditional overexpression was achieved using the binary UAS/Gal4 system in combination with the thermo-sensitive Gal80^ts^ inhibitor. The *pannier*-Gal4 driver (*pnr-Gal4*), *engrailed*-Gal4 (*en-Gal4*) and *scalloped*-Gal4 (*sd-Gal4*) drivers were used.

### Immunostainings and Microscopy

*Drosophila* nota and wing imaginal discs were dissected from staged pupae and larvae and stained using standard techniques. Primary antibodies were: goat anti-GFP (1∶500; ab5450 from abcam), guinea-pig anti-Senseless (1∶3000; kind gift from H. Bellen), mouse anti-Cut 2B10 mAb (1∶500; DSHB), rat anti-Elav 7E8A10 mAb (1∶100; DSHB). Secondary antibodies were from Jackson ImmunoResearch Laboratories and coupled to Cy2, Cy3 and Cy5. Wing discs and nota were mounted in Mowiol 4–88 **(**Sigma) containing 2,5% DABCO (Sigma). Images were acquired using a Leica SPE confocal microscope using 20x (HCX PL APO CS, NA 0.6) and 63x (HCX PL APO CS, N.A. 1.3) objectives. Adult flies were imaged using a Zeiss Discovery V20 stereo-macroscope using a 1.0X (PlanApo S FWD 60 mm) objective. Images were processed using ImageJ and Adobe Photoshop softwares.

## Supporting Information

Figure S1 **Sequence alignment of Insb proteins.** Multiple sequence alignment analysis revealed two regions conserved between different *Drosophila* species and an amino-terminal nuclear localization signal. Identical (red), similar (blue) and variable (black) amino acids are color-coded.
(TIF)

## References

[1] Artavanis-Tsakonas S, Rand MD, Lake RJ (1999) Notch signaling: cell fate control and signal integration in development. Science 284: 770–776. 10221902 10.1126/science.284.5415.770

[2] Kopan R, Ilagan MX (2009) The canonical Notch signaling pathway: unfolding the activation mechanism. Cell 137: 216–233. 19379690 10.1016/j.cell.2009.03.045 PMC2827930

[3] Morel V, Schweisguth F (2000) Repression by suppressor of hairless and activation by Notch are required to define a single row of single-minded expressing cells in the Drosophila embryo. Genes Dev 14: 377–388. 10673509 PMC316365

[4] Koelzer S, Klein T (2003) A Notch-independent function of Suppressor of Hairless during the development of the bristle sensory organ precursor cell of Drosophila. Development 130: 1973–1988. 12642500 10.1242/dev.00426

[5] Castro B, Barolo S, Bailey AM, Posakony JW (2005) Lateral inhibition in proneural clusters: cis-regulatory logic and default repression by Suppressor of Hairless. Development 132: 3333–3344. 15975935 10.1242/dev.01920

[6] Furriols M, Bray S (2000) Dissecting the mechanisms of suppressor of hairless function. Dev Biol 227: 520–532. 11071771 10.1006/dbio.2000.9923

[7] Kao HY, Ordentlich P, Koyano-Nakagawa N, Tang Z, Downes M, et al (1998) A histone deacetylase corepressor complex regulates the Notch signal transduction pathway. Genes Dev 12: 2269–2277. 9694793 10.1101/gad.12.15.2269 PMC317043

[8] Oswald F, Winkler M, Cao Y, Astrahantseff K, Bourteele S, et al (2005) RBP-Jkappa/SHARP recruits CtIP/CtBP corepressors to silence Notch target genes. Mol Cell Biol 25: 10379–10390. 16287852 10.1128/MCB.25.23.10379-10390.2005 PMC1291242

[9] Castel D, Mourikis P, Bartels SJ, Brinkman AB, Tajbakhsh S, et al (2013) Dynamic binding of RBPJ is determined by Notch signaling status. Genes Dev 27: 1059–1071. 23651858 10.1101/gad.211912.112 PMC3656323

[10] Mulligan P, Yang F, Di Stefano L, Ji JY, Ouyang J, et al (2011) A SIRT1-LSD1 corepressor complex regulates Notch target gene expression and development. Mol Cell 42: 689–699. 21596603 10.1016/j.molcel.2011.04.020 PMC3119599

[11] Dou S, Zeng X, Cortes P, Erdjument-Bromage H, Tempst P, et al (1994) The recombination signal sequence-binding protein RBP-2N functions as a transcriptional repressor. Mol Cell Biol 14: 3310–3319. 8164682 10.1128/mcb.14.5.3310-3319.1994 PMC358697

[12] Hsieh JJ, Zhou S, Chen L, Young DB, Hayward SD (1999) CIR, a corepressor linking the DNA binding factor CBF1 to the histone deacetylase complex. Proc Natl Acad Sci U S A 96: 23–28. 9874765 10.1073/pnas.96.1.23 PMC15086

[13] Yatim A, Benne C, Sobhian B, Laurent-Chabalier S, Deas O, et al (2012) NOTCH1 nuclear interactome reveals key regulators of its transcriptional activity and oncogenic function. Mol Cell 48: 445–458. 23022380 10.1016/j.molcel.2012.08.022 PMC3595990

[14] Krejci A, Bray S (2007) Notch activation stimulates transient and selective binding of Su(H)/CSL to target enhancers. Genes Dev 21: 1322–1327. 17545467 10.1101/gad.424607 PMC1877745

[15] Schweisguth F, Posakony JW (1992) Suppressor of Hairless, the Drosophila homolog of the mouse recombination signal-binding protein gene, controls sensory organ cell fates. Cell 69: 1199–1212. 1617730 10.1016/0092-8674(92)90641-o

[16] Nagel AC, Krejci A, Tenin G, Bravo-Patino A, Bray S, et al (2005) Hairless-mediated repression of notch target genes requires the combined activity of Groucho and CtBP corepressors. Mol Cell Biol 25: 10433–10441. 16287856 10.1128/MCB.25.23.10433-10441.2005 PMC1291231

[17] Bang AG, Hartenstein V, Posakony JW (1991) Hairless is required for the development of adult sensory organ precursor cells in Drosophila. Development 111: 89–104. 2015800 10.1242/dev.111.1.89

[18] Brou C, Logeat F, Lecourtois M, Vandekerckhove J, Kourilsky P, et al (1994) Inhibition of the DNA-binding activity of Drosophila suppressor of hairless and of its human homolog, KBF2/RBP-J kappa, by direct protein-protein interaction with Drosophila hairless. Genes Dev 8: 2491–2503. 7958912 10.1101/gad.8.20.2491

[19] Bang AG, Posakony JW (1992) The Drosophila gene Hairless encodes a novel basic protein that controls alternative cell fates in adult sensory organ development. Genes Dev 6: 1752–1769. 1516831 10.1101/gad.6.9.1752

[20] Morel V, Lecourtois M, Massiani O, Maier D, Preiss A, et al (2001) Transcriptional repression by suppressor of hairless involves the binding of a hairless-dCtBP complex in Drosophila. Curr Biol 11: 789–792. 11378391 10.1016/s0960-9822(01)00224-x

[21] Schweisguth F, Lecourtois M (1998) The activity of Drosophila Hairless is required in pupae but not in embryos to inhibit Notch signal transduction. Dev Genes Evol 208: 19–27. 9518521 10.1007/s004270050149

[22] Bailey AM, Posakony JW (1995) Suppressor of hairless directly activates transcription of enhancer of split complex genes in response to Notch receptor activity. Genes Dev 9: 2609–2622. 7590239 10.1101/gad.9.21.2609

[23] Zheng L, Carthew RW (2008) Lola regulates cell fate by antagonizing Notch induction in the Drosophila eye. Mech Dev 125: 18–29. 18053694 10.1016/j.mod.2007.10.007 PMC2782576

[24] Rouault H, Mazouni K, Couturier L, Hakim V, Schweisguth F (2010) Genome-wide identification of cis-regulatory motifs and modules underlying gene coregulation using statistics and phylogeny. Proc Natl Acad Sci U S A 107: 14615–14620. 20671200 10.1073/pnas.1002876107 PMC2930411

[25] Duan H, Dai Q, Kavaler J, Bejarano F, Medranda G, et al (2011) Insensitive is a corepressor for Suppressor of Hairless and regulates Notch signalling during neural development. EMBO J 30: 3120–3133. 21765394 10.1038/emboj.2011.218 PMC3160191

[26] Dai Q, Andreu-Agullo C, Insolera R, Wong LC, Shi SH, et al (2013) BEND6 is a nuclear antagonist of Notch signaling during self-renewal of neural stem cells. Development 140: 1892–1902. 23571214 10.1242/dev.087502 PMC3631965

[27] Venken KJ, He Y, Hoskins RA, Bellen HJ (2006) P[acman]: a BAC transgenic platform for targeted insertion of large DNA fragments in D. melanogaster. Science 314: 1747–1751. 17138868 10.1126/science.1134426

[28] Venken KJ, Carlson JW, Schulze KL, Pan H, He Y, et al (2009) Versatile P[acman] BAC libraries for transgenesis studies in Drosophila melanogaster. Nat Methods 6: 431–434. 19465919 10.1038/nmeth.1331 PMC2784134

[29] Gho M, Bellaiche Y, Schweisguth F (1999) Revisiting the Drosophila microchaete lineage: a novel intrinsically asymmetric cell division generates a glial cell. Development 126: 3573–3584. 10409503 10.1242/dev.126.16.3573

[30] Graveley BR, Brooks AN, Carlson JW, Duff MO, Landolin JM, et al (2011) The developmental transcriptome of Drosophila melanogaster. Nature 471: 473–479. 21179090 10.1038/nature09715 PMC3075879

[31] Parks AL, Cook KR, Belvin M, Dompe NA, Fawcett R, et al (2004) Systematic generation of high-resolution deletion coverage of the Drosophila melanogaster genome. Nat Genet 36: 288–292. 14981519 10.1038/ng1312

[32] Hartenstein V, Posakony JW (1990) A dual function of the Notch gene in Drosophila sensillum development. Dev Biol 142: 13–30. 2227090 10.1016/0012-1606(90)90147-b

[33] Micchelli CA, Rulifson EJ, Blair SS (1997) The function and regulation of cut expression on the wing margin of Drosophila: Notch, Wingless and a dominant negative role for Delta and Serrate. Development 124: 1485–1495. 9108365 10.1242/dev.124.8.1485

[34] Housden BE, Millen K, Bray SJ (2012) Drosophila Reporter Vectors Compatible with PhiC31 Integrase Transgenesis Techniques and Their Use to Generate New Notch Reporter Fly Lines. G3 (Bethesda) 2: 79–82. 22384384 10.1534/g3.111.001321 PMC3276196

[35] Bang AG, Bailey AM, Posakony JW (1995) Hairless promotes stable commitment to the sensory organ precursor cell fate by negatively regulating the activity of the Notch signaling pathway. Dev Biol 172: 479–494. 8612965 10.1006/dbio.1995.8033

[36] Furriols M, Bray S (2001) A model Notch response element detects Suppressor of Hairless-dependent molecular switch. Curr Biol 11: 60–64. 11166182 10.1016/s0960-9822(00)00044-0

[37] Lecourtois M, Schweisguth F (1995) The neurogenic suppressor of hairless DNA-binding protein mediates the transcriptional activation of the enhancer of split complex genes triggered by Notch signaling. Genes Dev 9: 2598–2608. 7590238 10.1101/gad.9.21.2598

