# Supplementary figures and images for: Insensible Is a Novel Nuclear Inhibitor of Notch Activity in Drosophila
Source: PLoS One. 2014 Jun 5;9(6):e98213. doi: 10.1371/journal.pone.0098213 (PMC4046977; doi:10.1371/journal.pone.0098213)

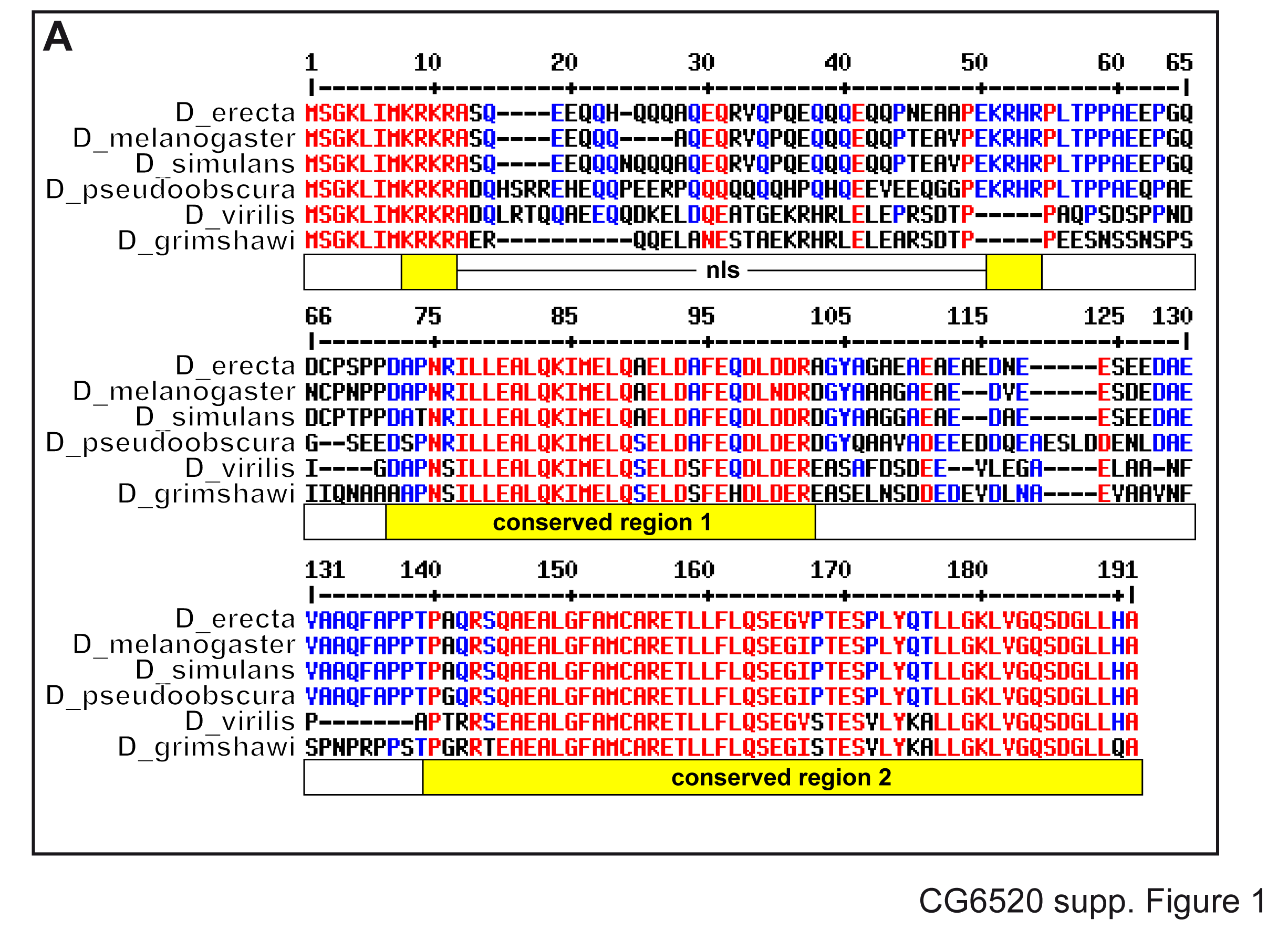

Supplement: Figure S1 — Sequence alignment of Insb proteins. Multiple sequence alignment analysis revealed two regions conserved between different Drosophila species and an amino-terminal nuclear localization signal. Identical (red), similar (blue) and variable (black) amino acids are color-coded. (TIF) [file pone.0098213.s001.tif]
